# A 20-Year-Old Man with IgA Vasculitis following COVID-19 Vaccination

**DOI:** 10.1155/2023/9505383

**Published:** 2023-07-17

**Authors:** Abdulaziz Alsubaie, Abdulmajeed Alshabanat, Abdulrahman Almizel, Mohammed Omair, Rahaf Alodaini

**Affiliations:** ^1^King Saud University College of Medicine, Department of Infectious Diseases, Riyadh, Saudi Arabia; ^2^King Saud University College of Medicine, Department of Internal Medicine, Riyadh, Saudi Arabia; ^3^Department of Medicine, McGill University, Montreal, QC, Canada

## Abstract

IgA vasculitis is a common type of vasculitis that is generally triggered by infectious causes. Vaccines have been reported as a trigger as well. Herein, we report a case of a young man who is previously healthy and who developed IgA vasculitis after the first dose of the COVID-19 mRNA vaccine Pfizer-BioNTech. The patient's symptoms were mainly skin and joint without renal or other system involvement. The patient had an excellent outcome with complete resolution after treatment with steroid tapering and azathioprine as a steroid-sparing agent over 6 months.

## 1. Introduction

IgA-associated vasculitis (formerly known as Henoch–Schonlein purpura) is a common type of vasculitis that is mediated by immune-complex deposition in the affected organ [1]. IgAV is characterized by skin, joints, gastrointestinal tract, and renal system involvement. Although the actual mechanism is unknown, different triggers have been reported including upper respiratory tract infection and vaccination like the measles-mumps-rubella (MMR) vaccine [2–4]. The diagnosis of IgAV is usually made clinically. However, a skin biopsy might be helpful in confirming the diagnosis. Kidney biopsy plays an important role when the renal system is involved [5, 6]. The prognosis is usually excellent in the pediatric population. However, there are more incidences of renal involvement and worse prognosis in the adult population [7]. IgAV is usually a self-limited disease with spontaneous recovery that requires only supportive measures to ease the pain and edema. However, systemic glucocorticoid and immunosuppressant agents might be added based on the severity.

## 2. Case Presentation

**Figure 1:**
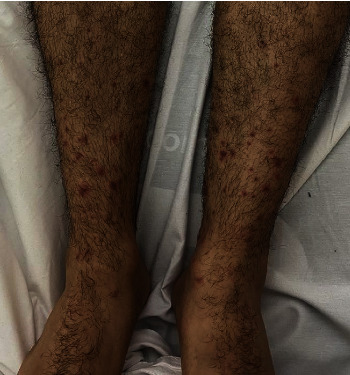
Bilateral nonblanching palpable purpuric rash in the lower extremities below the knee joint.

**Figure 2:**
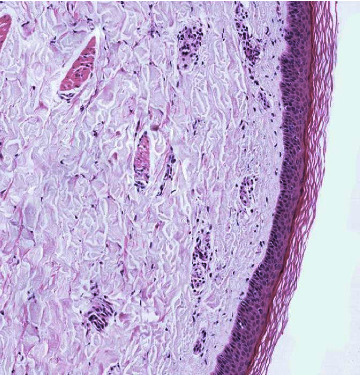
Skin punch biopsy showing perivascular lymphocytic infiltrates.

Here, we present a 20-year-old gentleman presented to the Emergency Department of a tertiary teaching hospital in Riyadh city, with complaints of right knee joint pain and swelling, and lower extremities rash. He was in his usual state of health until two weeks prior to his presentation when he received the COVID-19 vaccine (first dose, Pfizer-BioNTech). Two days after, he developed two episodes of diarrhea, nonbloody and not associated with other gastrointestinal symptoms, which resolved spontaneously. Four days after the vaccine, he started to have pain in his right knee, and he noticed swelling that is progressive in nature, which interfering with daily activities such as walking. He also noticed a rash in his lower extremities. He denied a history of fever, genito-urinary symptoms, personal or family history of autoimmune diseases, previous sexual encounters, or drug use. His immunizations were up to date. His physical examination was remarkable for a nonblanching bilateral palpable purpuric rash in the lower extremities below the knee joint (Figure 1). A moderate right knee swelling is observed associated with painful and limited passive and active flexion and extension. Other systems including genial examination were unremarkable. Laboratory results revealed a normal complete blood count, liver function test, renal function test, electrolytes, and coagulation panel. Antinuclear antibody, antidouble-stranded DNA antibody, and rheumatoid factor were all negative. C3 and C4 levels were normal while the erythrocyte sedimentation rate (ESR) was high at 48 millimeters/hour, and the serum IgA was mildly elevated at 3.71 g/L. Human immunodeficiency virus antigen/antibody testing (combo test) was negative as well as hepatitis B surface antigen and hepatitis C PCR. Urinalysis was also negative for red and white blood cells, and there was no proteinuria. Stool analysis was positive for occult blood. Skin biopsy showed features suggestive of small vessel vasculitis on histopathology (Figure 2). Immunofluorescence studies showed moderate IgA positivity in the walls of some blood vessels. C3, IgG, and IgM were negative. The patient was discharged with a near-term follow-up. However, 7 days after the discharge, he started to experience severe abdominal pain, diffuse, constant in nature, not responding to simple analgesia, requiring him to present to the emergency department. On examination, the abdomen was soft with no peritoneal signs, the abdominal film was unremarkable, and the computed tomography of the abdomen was normal. He was started on oral prednisolone 30 mg and azathioprine with significant improvement of his symptoms and rash.

## 3. Discussion

IgA vasculitis is a small vessel disease characterized by the deposition of IgA antibodies and triggers such as infections and drugs have been documented in the literature. In our case, the patient's trigger was most likely the COVID-19 vaccine based on his history and physical exam, along with the laboratory results and skin findings and the timing of the COVID-19 vaccine. HSP is a form of IgA that is most common in children, the presentation can be variable, and the etiology is not well studied but believed to be multifactorial. It has been reported after vaccination in multiple case reports (influenza, HAV, HPV, etc.), and now, the massive vaccination of COVID-19 is a possible increase in such cases to be seen either reactivation of a known patients with IgA vasculitis or a development of a new diagnosis. The mechanism of such reactivation or de novo IgA vasculitis is unclear; however, it is thought to be linked to the development anti-SARS-2 IgA against the COVID-19 spike protein and IgA vasculitis [8]. The aim of our report is to highlight the possible association and to review the potential management and complication if any. The following Table 1 is a summary of previously published case reports describing vaccine-associated IgA vasculitis. In most of the reports, it was treated with short courses (3–7 days) of low-dose steroids (prednisolone <10 mg/day) with significant improvement. Some reports used longer periods and higher doses depending on the severity as there are no specific guidelines or consensus to treat such cases. In our case, due to relapse and readmission, he was started on a higher dose of prednisolone 30 mg oral daily and azathioprine 100 mg oral daily, with resolution of his condition and without recurrence.

## Figures and Tables

**Table 1:** Summary of published case reports.

Author	Year	Vaccine	Time of onset	Biopsy/IF result	Treatment	Outcome
Lambert et al. [9]	2003	Meningococcal polysaccharide vaccine	7 Days	Leukocytoclastic vasculitis	Steroid taper	Mild nephropathy
Jariwala et al. [10]	2011	HAV vaccine	4 hours	Not performed^*∗*^	Supportive	Resolution with no recurrence
Gomes et al. (2 cases) [11]	2013	HPV vaccine	3 Days after the second dose of the HPV vaccine	Leukocytoclastic vasculitis. IF negative for IgA deposition	Steroid taper with azathioprine	Resolution with no recurrence
Malek et al. [12]	2018	Influenza vaccine	2 Days	Not performed^*∗*^	Short course of steroid	Resolution with no recurrence
Cohen et al. [13]	2021	Pfizer-BioNTech	2 Days	Leukocytoclasia and erythrocytes extravasation	Steroid taper	Stabilization of her primary disease after steroid
Hines et al. [14]	2021	Pfizer-BioNTech	2 Days	Not performed^*∗*^	Supportive	Rash resolved with no recurrence

^*∗*^Due to the strong clinical suspicion.
